# Postural control as a function of time-of-day: influence of a prior strenuous running exercise or demanding sustained-attention task

**DOI:** 10.1186/1743-0003-10-26

**Published:** 2013-03-01

**Authors:** Thibault Deschamps, Justine Magnard, Christophe Cornu

**Affiliations:** 1 University of Nantes, Laboratory “Motricité, Interactions, Performance” (EA 4334), F-44000, Nantes, France

**Keywords:** Posture, Attention, Exercise, Time-of-day

## Abstract

**Background:**

The current experiment investigated the impact of two potential confounding variables on the postural balance in young participants: the induced-experimental activity prior to the static postural measurements and the well-documented time-of-day effects. We mainly hypothesized that an exhaustive exercise and a high attention-demanding task should result in alterations of postural control.

**Methods:**

Ten participants performed three experimental sessions (differentiated by the activity – *none*, *cognitive* or *physical* – prior of the assessment of postural stability), separated by one day at least. Each session included postural balance assessments around 8 a.m., 12.00 p.m. and 5 p.m. ± 30 min. The physical and cognitive activities were performed *only* before the 12 o’clock assessment. The postural tests consisted of four conditions of quiet stance: stance on a firm surface with eyes open; stance on a firm surface with eyes closed; stance on a foam surface with eyes open and stance on a foam surface with eyes closed. Postural performance was assessed by various center of pressure (COP) parameters.

**Results:**

Overall, the COP findings indicated activity-related postural impairment, with an increase in body sway in the most difficult conditions (with foam surface), especially when postural measurements are recorded just after the running exercise (physical session) or the psychomotor vigilance test (cognitive session).

**Conclusions:**

Even if no specific influence of time-of-day on static postural control is demonstrated, our results clearly suggest that the activities prior to balance tests could be a potential confounding variable to be taken into account and controlled when assessing clinical postural balance.

## Introduction

Many studies have already looked at the time-of-day dependence of postural parameters [1,2] and the effect of physical activity on human postural control, see [3] for a review. Even if typically dual-task performances have been extensively studied (evidencing that quiet standing requires cognitive resources, [4,5]), the novelty of the present experiment resides in studying the effects of a psychomotor vigilance test prior to the balance tests in young people. Apart from the fact of questioning the clinical utility of posturography [6], the reliability of various postural measurements (derived from the center of pressure – COP) [7] and the subsequent methodological recommendations [8], the determination of confounding variables (aging, gender, sleep deprivation…) (e.g. [9,10]) when assessing postural balance in people is a major matter to be investigated. If postural instability increases as a function of (demanding physical and/or cognitive) activities prior to postural measurements, the relevance for using (clinical) information coming from the evaluation of postural stability should have to be raised. In this respect, the confounding variables must be clearly identified and controlled. Thus the purposes of the present study are twofold: first, it aims to confirm time-of-day effects on postural stability [1,2] for young subjects; secondly, it explores the impact of an induced-experimental activity (i.e. an exhaustive physical exercise *or* a sustained-attention task *or* no specific activity) prior to the assessment of postural stability. To our knowledge, no study has yet investigated these potential confounding variables at the same time.

Among the numerous well-documented confounding variables when performing force plateform balance tests, two important categories of systems are of overriding interest: the neuromuscular system (and fatigue due to muscular exercise) and the cognitive system (and mental fatigue due to a demanding sustained-attention task). Concerning the effects of neuromuscular constraints on postural control, a considerable number of studies have demonstrated that both general and local exercises (such as walking, running, cycling, the repetition of simple segmental movements, or maintenance of isometric (or dynamic) muscular actions) contribute to alter the effectiveness of sensory inputs and motor output of postural control (see [3], for a recent review). The duration of postural disturbance after general (or local) muscular exercise was found to be relatively short (about 10–20 min.) [11-13]. Additionally, according to the context of muscle fatigue, different compensatory postural strategies (e.g. sensory compensation, [11]) have been identified to counteract or limit the balance control impairment. For example, Simoneau *et al.*[14] confirmed the immediate decrement of the postural performance (COP displacements) measured just after moderate fatigue (experimentally induced by three periods of fast walking on a treadmill), but their participants were able to quickly compensate for this effect of moderate fatigue by a higher cognitive resources investment necessary to maintain active postural control [5,15].

With respect to cognitive constraints, consistent findings underlined the importance of considering the interplay of cognitive load (or attentional resources investment) and stability of motor performance [16-18]. Specifically, if postural control has often been thought of as an automatic- or reflexively-controlled task, clear evidence of attentional processing requirements to postural control have been discovered (e.g. [19,20]). Using typically the dual-task methodology, numerous studies have demonstrated that attentional demands associated with balance control have a dependence on the complexity of the dual postural tasks [5,21]. In more challenging conditions (such as standing on a foam surface with eyes closed – no regulation of visual inputs is available), postural tasks are more cognitively demanding [4,14]. More precisely, the extent to which the performance on either task declines indicates the extent to which the two tasks share attentional resources, as evidence of cognitive and specifically attentional allocation deficits. In the logic of this attentional depletion, we thus emphasise the impact of cognitive fatigue due to a sustained-attention task [22] experimentally induced before postural performance measurements. Recent studies have showed that the performance of demanding tasks during extensive periods of time leads to cognitive resource allocation deficits and a decrease in subsequent performance [18,23,24].

Apart from the effects of attentional resource depletion over time on motor performance stability [23], we also aim to isolate any time-of-day effect on postural control (e.g. [25]), a clinical factor to be controlled when assessing postural balance [2]. For example, Gribble *et al.*[1] tested young people (mean age: 21.8) on static and dynamic postural tasks at 10:00, 15:00, and 20:00. Overall, their results indicate that performance in postural control tasks was better in the morning than in the afternoon or evening. With older adults, Jorgensen *et al.*[2] have recently confirmed the importance for controlling the time of day, with a main significant variation of postural balance between 9 a.m., 12:30 p.m., and 4 p.m. (poorer balance control in the afternoon).

### Aims of the study and hypotheses

In this experiment, we aimed to characterize the postural balance of young participants as a function of the induced-experimental activity prior to the static postural measurements. We hypothesized that an exhaustive exercise and a high attention-demanding task should result in alterations of postural control. Concerning the time-of-day variable, we suggested that the well-documented effects of afternoon on postural control could be intensified due to the previous specific activities.

## Methods

### Participants and apparatus

Ten male students from the faculty of Sport Sciences (University of Nantes) aged 22.1 ± 1.7 years (mean ± SD) (height = 176.1 ± 4 cm, weight = 71 ± 7.1 kg) volunteered to participate in the present study. Participants exhibited no visual (or corrected vision) or physical impairment. The experiment was undertaken with the understanding and written consent of each participant, and was conducted according to the Helsinki Statement (1964).

For all data collection of postural sway, we used a Kistler force plateform (model 9286BA) with subject weight normalization. Data were sampled at 100 Hz and recorded online on a personal computer.

### Procedure

Participants performed three experimental sessions (differentiated by the activity – none, cognitive or physical – prior of the assessment of postural stability), separated by at least one day. Each session included measurements around 8 a.m., 12:00 p.m. and 5 p.m. ± 30 min. The physical and cognitive activities were performed *only* before the 12 o’clock assessment. The three sessions were randomly counterbalanced between participants.

For each measurement time, the postural test consisted of four conditions of quiet stance: stance on a firm surface with eyes open (EO); stance on a firm surface with eyes closed (EC); stance on a foam surface (thickness 7 cm) with eyes open (FEO); and stance on a foam surface with eyes closed (FEC). Participants stood quietly while barefoot, with the head in a straight-ahead position, their arms along the body. During conditions with eyes open, they were instructed to look a black spot (with a diameter 2 cm) placed in white wall in the front at a 2 m distance. For each condition, three trials were performed. The duration of each trial was 90 s, followed by a short rest period. The twelve trials were presented randomly. The whole experiment time was about 25 min. Under these recommended conditions (three trials, sampling duration of 90 s, sampling frequency of 100 Hz and cut-off frequency of 10 Hz, visual and surface conditions), the high reliability of COP parameters has previously been reported in the literature (see [8] for a noticeable review). Thus the results have been averaged across trials.

In the physical session (S_phys_), around 11.15 a.m. (± 15 min), participants were required to perform a set of sprints (40 meters of distance) over 40 min. Rest periods of 45 s were given between each run. Thus the number of sprints performed by each participant was about 44. In order to validate the impact of this exhausting running exercise, a functional vertical jumping test was performed just before and after the running session. Participants were required to perform five consecutive countermovement vertical jumps, with instructions to reach and maintain this target maximal height for each jump. No time constraint was imposed. The jumping height was obtained using an infrared timing system (Optojump, Microgate SRL, Rome, Italy).

During the cognitive session (S_cog_), the psychomotor vigilance test [26] was used around 11.15 a.m. (± 15 min) as a sustained-attention task prior to the postural tests. Thus we used the simple reaction time (RT) test with varying and random inter-stimulus intervals ranging from 2 to 10 s (including a 1 s delay after their response for participants to read their reaction time). Similar to [22], participants were instructed to focus their attention on a red, rectangular box in the middle of a black screen, and monitor the space for the appearance of a millisecond counter stimulus. They were asked to react as quickly as possible to this stimulus by pressing the space button of a keyboard. In order to elicit a greater time-on-task effect and (almost) match the session’s duration, the participants underwent two bouts of a psychomotor vigilance test of 15 min duration, separated by a short rest period (1–3 min). The time interval between the physical / cognitive intervention and the start of the balance recordings was of 5 minutes.

For the control session (S_cont_), no specific test prior to the noon postural test was carried out. Additionally, they were asked not to exercise during the day.

### Data and statistical analysis

For measuring all of the COP parameters, raw data were filtered using a fourth-order Butterworth, zero-phase low-pass at 10 Hz. Then the COP parameters were standard deviation (SD) of amplitude in antero-posterior (AP) and medio-lateral (ML) directions, SD of velocity in AP and ML directions, mean velocity and area (95% confidence ellipse) (see [27] or [7] for details of calculus formula). For testing the effects of prior activity, time-of-day and visual/ surface conditions on the postural control, different analyses of variance (ANOVAs) with repeated measures were carried out for each aforesaid dependent variable. The first 3 (Session) × 4 (Condition) ANOVA was performed to verify that all the participants produce the same level of postural instability at 8 a.m. We expected no main effect for session and no interaction involving session. A second ANOVA with 3 (Session) × 3 (Time-of-Day) × 4 (Condition) within-participants factors was performed. For each analysis, the level of significance was p < 0.05. Least Significant Difference comparisons were used for post-hoc tests following significant effects. If the sphericity assumption in repeated measures ANOVA was violated (Mauchly’s test), the corrected tests of significance were used [28,29]. In that case, the paired *t*-tests with corrected alpha level were used as post-hoc comparisons.

From each psychomotor vigilance test (PVT), we extracted the following variables as a measure of overall level of performance: mean (SD) reaction time (RT), and number of lapses (RT > 500 ms). All RT values below 100 ms (considered as anticipated, see [30]) were removed from the data. Considering all participants and all conditions, 0.15% of the *RT*s were discarded. To assess the time-on-task effect, we divided the PVT bout into 6-min quintiles and obtained the mean RT in each of those time bins, as well as computed the percentage change in reaction times from the first to the last quintile for each subject. One-way analysis of variance (within subject repeated measures) of mean RT in the 6-min quintiles was performed. *LSD* comparisons were used for post hoc tests when significant effects were identified.

For each vertical jumping test, we extracted the minimal and the maximal, and the mean height (in cm) of five consecutive jumps. Unfortunately, only six participants’ performances were recorded because of technical problems with the infrared timing system. For each of these dependent variables, a paired *t*-test was carried out. In the following section, only the significant results are presented.

## Results

### Reaction time

**Figure 1:**
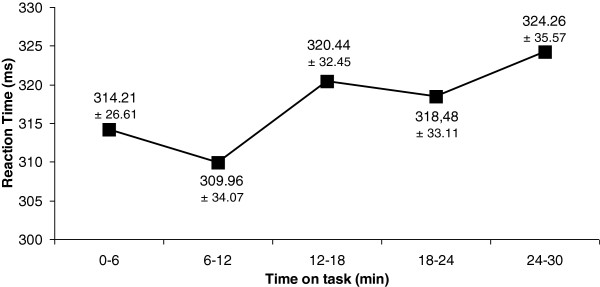
Means and standard deviations of reaction time from the first to the last 6-min quintiles of the 30-min psychomotor vigilance test.

Mean (SD) RT (317.46 ± 31.71 ms) to the PVT were computed for all subjects. Participants were attentive to the task, as shown by the relatively small number of lapses committed overall (mean = 3.9 SD = 2.64). The analysis revealed a significant main effect of time-on-task [*F*(4, 45) = 3.73, *p* < 0.05]. LSD’s post-hoc comparisons showed significant differences between RT in the first and last quintile (p < 0.01), and the second and three last quintiles (p < 0.05). Performing the PVT elicited a clear time-on-task effect, with most participants showing steadily increasing RT over the course of the PVT (Figure 1). Time-on-task vulnerability was quantified by calculating the percentage change in mean reaction times from the first to last quintile of the PVT for each subject. These values ranged from −5.19% to 11.34%. There were noticeable inter-individual differences in the extent of this vulnerability.

### Jumping height

The Student’s *t*-test for the jumping mean height indicated a significant difference (*t*(5) = 4.74; p < 0.01) between the performance before (38.48 ± 3.46 cm) and just after (31.95 ± 5.08 cm) the running test in physical session. Identical results have been revealed by the *t*-tests for the minimum and maximal height variables (*t*(5) = 3.79; p < 0.05 and *t*(5) = 4.35; p < 0.01 respectively). Performing an exhaustive effort over 40 min caused a clear impairment of participant physical abilities, with probable fatigue mainly localized in the lower limbs.

### Postural instability

**Figure 2:**
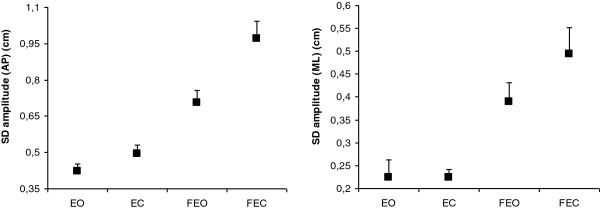
**Effects of Condition on standard deviation (SD) of COP amplitude (cm) in antero-posterior (AP) and medio-lateral (ML) directions, when measured at 8.00 a.m.** Error bars correspond to the interindividual variability (standard deviation).

We first focus on the postural performance considering only the measures collected at 8.00 a.m. All statistical results are summarized in Table 1. Overall, the analysis concerning all COP parameters revealed a main effect of Condition, with significant alterations of postural control under compliant conditions (FEO, FEC) compared to firm conditions (EO; EC). Additionally, post-hoc comparisons showed that the postural instability in FEC was higher than in FEO, as illustrated by Figure 2 for standard deviation of the COP in AP and ML directions. No effect of Session or no interaction involving this factor was revealed.

**Table 1:** Analysis of variance results (F values) for all the COP parameters for the different factors

	**Session**	**Condition**	**Session × Condition**
**COP parameters**	(2, 18)	(3, 27)	(6, 54)
SD amplitude (AP) (cm)	0,2	113,4***	0,9
SD velocity (AP) (cm/s)	2,8	100***	1,6
SD amplitude (ML) (cm)	1,7	108***	1,5
SD velocity (ML) (cm/s)	0,03	27,47***	1,3
Mean Velocity (cm/s)	0,2	147,4***	0,9
Area (95% ellipse) (cm^2^)	1,6	179,4***	0,9

Note. Factors were Session and Condition. Degrees of freedom are shown in parentheses. COP: center of pressure; AP: anteroposterieur; ML: mediolateral.

*** *p* < 0.001.

Concerning the second ANOVA with 3 (Session) × 3 (Time-of-Day) × 4 (Condition) between-participants factors, all statistical results are summarized in Table 2.

**Table 2:** Analysis of variance results (F values) for all the COP parameters for the different factors

	**S**	**TD**	**C**	**S × TD**	**S × C**	**TD × C**	**S × TD × C**
**COP parameters**	(2, 18)	(2, 18)	(3, 27)	(4, 36)	(6, 54)	(6, 54)	(12, 108)
SD amplitude (AP)	0,6	5,9*	181,4***	0,9	2,5*	1,7	1,1
SD velocity (AP)	6,54**	3,5*	75,27***	0,59	2,64*	1,91	0,56
SD amplitude (ML)	3,35	8,03**	89,18***	2,24	1,18	2,57*	1,74
SD velocity (ML)	1,47	1,65	48,2***	1,44	2,64*	1,03	0,73
Mean Velocity	1,2	4,3*	110,8***	1,5	1,1	2	0,7
Area (95% ellipse)	3,76*	8,06**	99,49*	1,89	2,77*	3,32**	2,17*

Note. Factors were Session, S, Time-of-Day, TD, and Condition, C. Degrees of freedom are shown in parentheses. COP: center of pressure; AP: anteroposterieur; ML: mediolateral.

* *p* < 0.05. ** *p* < 0.01. *** *p* < 0.001.

**Figure 3:**
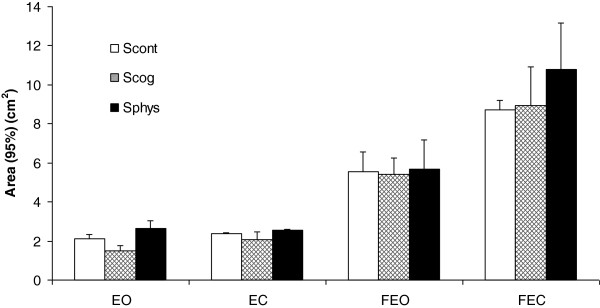
**Means of 95% ellipse area (cm2) for eyes open (EO), eyes closed (EC), foam eyes open (FEO) and foam eyes closed (FEC) postural conditions, as a function of session (S**_**cont**_**: control session; S**_**cog**_**: cognitive session; S**_**phys**_**: physical session).** Error bars correspond to the interindividual variability (standard deviation).

Firstly, it is worth noting that the main effect of Condition is systematically revealed by the analysis, with recurrent evidence of higher postural degradation in compliant conditions than for firm conditions. More interestingly are the results observed for the area parameter, which appears the most relevant and sensitive – in this present case - to experimental factors and their combinations: all main effects and interactions (except one) were significant. In support of our study hypothesis, confidence ellipse area (cm^2^) was significantly larger in the S_phys_ (5.4 ± 0.48) than in the S_cog_ (4.47 ± 0.29) and in the S_cont_ (4.68 ± 0.41). No area difference between S_cog_ and S_cont_ is observed (p = 0.56). The findings are confirmed by a Session × Condition interaction. Post-hoc analysis showed significant differences between the S_phys_ (10.77 ± 2.38) and S_cog_ (8,94 ± 1.97) (p < 0.05), and between the S_phys_ and S_cont_ (8,5 ± 0.5) only for FEC (Figure 3).

**Figure 4:**
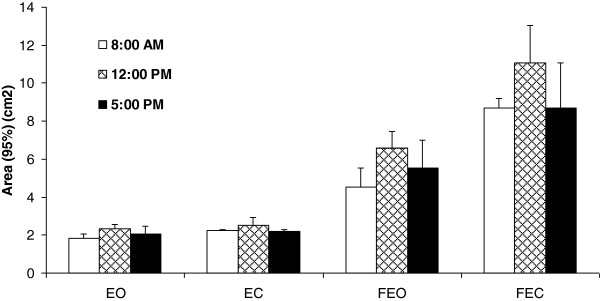
**Means of 95% ellipse area (cm2) for eyes open (EO), eyes closed (EC), foam eyes open (FEO) and foam eyes closed (FEC) postural conditions, as a function of time-of-day.** Error bars correspond to the interindividual variability (standard deviation).

Secondly, the analysis yielded a significant effect of Time-of-Day, with a larger confidence ellipse area in mid-day (5.62 ± 3.88) relative to the morning (4.33 ± 2.85) and the afternoon (4.61 ± 2.94). Moreover, the significant Time-of-Day × Condition confirmed larger areas around 12.00 p.m. as compared to recordings at 8 a.m. and 5 p.m., but only for the FEO and FEC conditions. No differences were found between the morning and the afternoon, whatever the condition (Figure 4).

**Figure 5:**
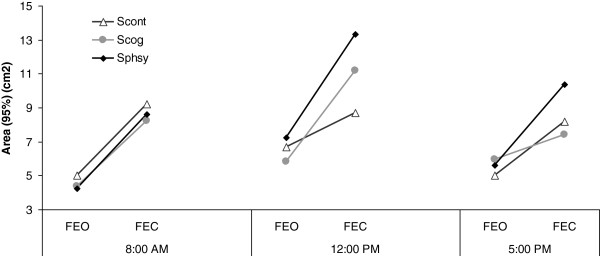
**Session × Time-of-Day × Condition interaction for area (cm2), only for foam eyes open (FEO) and foam eyes closed (FEC) conditions.** Note. S_cont_: control session; S_cog_: cognitive session; S_phys_: physical session.

Finally, ANOVA yielded a significant Session × Time-of-Day × Condition (p < 0.02). First, it should be noted that post-hoc comparisons localized differences only for the compliant conditions (shown in Figure 5). Considering *the FEC condition*, larger areas are found at 12 p.m. relative to 8 a.m. and to 5 p.m. during the S_cog_ and the S_phys_. Interestingly, the area is larger in the afternoon relative to the morning during the S_phys_ (no similar difference is observed during S_cog_). Besides, at 12 p.m., post-hoc comparisons indicated a larger area in S_phys_ than in the S_cog_. A significant lower area in S_cont_ is also observed as compared with the S_cog_. For *the FEO condition*, statistics yielded larger areas at 12 p.m. relative to 8 a.m. and to 5 p.m. only during the S_phys_ (no difference between 8 a.m. and 5 p.m.)

## Discussion

The purpose of the present study was to explore the impact of pre-exhaustive activities (a strenuous physical exercise *or* a sustained attention task) on the resulting postural stability. This latter was inferred from different COP parameters in four postural conditions. In support of our study hypothesis, the COP findings indicated activity-related postural impairment, with an increased significant body sway in the most difficult conditions (with foam surface), especially when postural measurements are recorded just after the running exercise or the psychomotor vigilance test (namely around mid-day). Even if no specific influence of time-of-day on static postural control is demonstrated (back down from this point below), our results clearly suggest that activities prior to balance tests could be a potential confounding variable to be taken into account and controlled when assessing clinical postural balance.

The first results to consider are those obtained during the physical and cognitive sessions, during the running exercise and the psychomotor vigilance test, respectively. Even if no physiological measurement was recorded to assess how exhausted the participants were after the running exercise, we can infer a localized muscle fatigue – or *a minima* alteration of participant’s neuromuscular capabilities in the lower limbs - induced by the repetition of intense sprints and from the jumping performance patterns [3]. As expected, participants were not able to jump as high after the sprints. Likewise, we replicated the results of [22] during the cognitive session when participants performed a simple reaction time test over 30 minutes. Consistently, participants reacted significantly slower to target stimuli as the task proceeded, which is evidence of fatigue in the neural attentional system [22]. Taken together, these expected results led to interpret of the COP parameters changes (especially at 12.00 p.m.) as a result of these exhaustion tasks on postural control. Lastly, considering the important methodological prerequisites for discussing the present findings, it is worth noting that the participants’ postural stability still was statistically equivalent at 8.00 a.m., whatever the session. Consequently, the postural balance changes observed at 12.00 p.m. or at 5.00 p.m. can be ascribed only to the time-of-day influences and/or the induced-experimental activities.

The increase of the COP parameters with altered proprioception (due to the standing on a foam surface) is not surprising. Additionally, further increase occurred in the absence of visual information (with eyes closed) in each session performed. Standing on a compliant surface alters the somatosensory information available for postural control and increases reliance on vestibular and visual information [31,32]. Thus, standing on a foam pad provided participants with a more challenging postural control task than simply standing on a firm flat surface. Crucially, this more challenging postural task was the most impacted during the physical session, certainly because of a decreased functionality of the proprioceptive muscle receptor system under fatigue e.g. [33]. Following peripheral and/or central muscle fatigue induced by exercise (e.g. running, cycling), an impairment of the proprioceptive and kinesthetic properties of joints is strongly hypothesized [34], induced by increasing the threshold of muscle spindle discharge, disrupting afferent feedback, and subsequently altering conscious joint awareness [35,36].

Considering postural balance measurements with previous physical or attentional loading, our results are consistent with the existing literature which documents the increase of postural instability with cognitive and important generalized metabolic fatigue-induced manipulations e.g. [37,38]. An interesting observation concerns the results at 12.00 p.m. At first glance, we indeed could suggest time-of-day effects on static postural balance control, but this assumption is quite inconsistent with [1] or [25]. In these studies with young adults, COP parameters were quite identical over a day or better (i.e. higher postural stability in static standing) in the morning than in the afternoon and evening. Alike in older adults, there is no significant difference between morning and mid-day in any of the sway parameters [2]. Thus, all time-of-day effects - always located at 12.00 p.m. – and some effects of the Session (see Table 2) really reflect the deleterious impact of a previous strenuous exercise or a high-attention-demanding task, irrespective of the time-of-measurement. The impact of an induced-experimental cognitive fatigue [22] prior to the assessment of postural performance is in line with the hypothesis of attentional resource depletion over time [18,23]. Certainly, the allocation of attention associated with the postural control is altered because of cognitive resource deficits [15]. Now, these results emphasize the importance of considering both physiological and psychological influences on motor performance [16,18] when clinically measuring human balance. Indeed this (to-be-tested) point may become more pronounced in older adults because of the required control of time-of-day when assessing postural balance [2].

In addition, we have shown for the first time that a fatiguing exercise prior to balance measurements induced effects on postural control over extensive periods, as evidenced by larger COP surface areas at 5.00 p.m. as compared with static postural balance measurements at 8.00 a.m. Consistent with the finding of our study, Bougard *et al.*[25] found no significant difference between the cognitive and control sessions. The postural control still is altered during the afternoon owing to the exhausting running exercise, which is quite different from the studies of [11] or [12]. Indeed they observed that the duration of postural disturbance after general muscular exercise is relatively short (about 10 to 20 min). Combined with the typical progression of muscle fatigue incurred by daily functions and work tasks [39], this strenuous exercise (of duration of 40 min) impacted the postural balance in the afternoon, by possibly impairing information from the vestibular, somatosensory or neuromuscular system (e.g. [40]).

## Conclusions

Our results show the importance of questioning the potential effects of daily fatiguing activities when evaluating age-related changes of human balance during quiet stance, especially in older adults [31]. This assumption is also confirmed by an increase in postural instability observed during the cognitive session at mid-day when compared with the control session. To the best of our knowledge, we are first to demonstrate the impact of cognitive fatigue on postural control in healthy young adults when experimentally induced prior to posturographic assessment. Similar to studies using a classical dual-task methodology (see [41] for a review) which also demonstrates that posture control and higher-level cognition have common resource requirements, these current findings may have strong scientific and clinical relevance when assessing postural balance in (older) people. Based on ample evidence suggesting that the decline in sensorimotor and cognitive function in older people adversely affects postural control (e.g. [4,42]), we indeed recommend integrating the influence of cognitive load induced by functional activities of daily life (which influence particular executive functions, [43]) in older adults, especially when sensory and neuromuscular adaptations are required [38]. Current research is using these results to determine the evaluative and predictive value of balance measurements when previous daily activities are directly controlled.

## Competing interests

The authors declare that they have no competing interests.

## Authors’ contributions

TD and JM participated in the conception and design of the experiment, performed the data acquisition, analyzed the data, drafted and revised the manuscript. CC conceived of the study and participated in the design and the coordination, and has made a substantial contribution drafting and critically revising the manuscript. All authors have read and approved the final manuscript.
